# Transarterial infusion chemotherapy combined with microwave ablation for inoperable triple-negative breast cancer: a case report

**DOI:** 10.3389/fonc.2026.1874496

**Published:** 2026-07-29

**Authors:** Yang Fang, Zhezhu Han, Haosen Xu, Yiming Zhang, Minghu Sun, Songnan Zhang

**Affiliations:** 1 Department of Oncology, The Affiliated Hospital of Yanbian University (Yanbian Hospital), Yanji, China; 2 Department of Interventional Radiology Center, The Affiliated Hospital of Yanbian University (Yanbian Hospital), Yanji, China

**Keywords:** case report, MWA, RECIST 1.1, TAI, TNBC

## Abstract

**Background:**

Early-stage triple-negative breast cancer is predominantly managed with multidisciplinary perioperative treatment strategies. However, the optimal therapeutic approach for elderly patients who are unsuitable for surgical resection and unable to tolerate systemic chemotherapy remains to be fully elucidated.

**Case presentation:**

We report the case of a patient diagnosed with triple-negative breast cancer who, following multidisciplinary team (MDT) evaluation, was considered unsuitable for surgical resection and unable to tolerate systemic chemotherapy. A locoregional treatment strategy was adopted. The patient received transarterial infusion chemotherapy with docetaxel (60 mg) and cisplatin (60 mg), followed two weeks later by ultrasound-guided microwave ablation of the breast lesion. Given the aggressive biological characteristics of triple-negative breast cancer, the large tumor size, and the use of local treatment, adjuvant local radiotherapy combined with oral capecitabine was administered for 6 months. Breast ultrasonography performed 3 months later demonstrated a marked reduction in lesion size (2.9 × 1.5 cm). After more than 3 years of follow-up, the patient maintained a partial response according to RECIST version 1.1 criteria, with the residual lesion measuring 2.4 cm and exhibiting well-defined margins.

**Conclusion:**

This case provides a potential alternative therapeutic option for patients with triple-negative breast cancer who are unsuitable for surgery and unable to tolerate systemic chemotherapy.

## Introduction

Triple-negative breast cancer (TNBC) is an aggressive breast cancer subtype associated with a high risk of early distant metastasis and adverse clinical outcomes (1, 2). Due to the absence of estrogen receptor, progesterone receptor, and human epidermal growth factor receptor 2 (HER2) expression, treatment options remain limited, and surgery combined with systemic chemotherapy remains the standard treatment for early-stage TNBC (3, 4). However, some patients are unable to tolerate surgery or systemic chemotherapy and lack effective alternative treatment options, often leading to rapid disease progression and adverse outcomes. Although immunotherapy has shown therapeutic potential in TNBC, low response rates and immune-related adverse events remain major challenges (5). Several studies have explored novel targeted therapies (6), yet an optimal treatment strategy has not been established. Therefore, exploring safe and effective treatment approaches for patients with TNBC who are unable to tolerate standard therapy remains clinically important.

Several studies have explored interventional therapies, including transarterial chemoembolization/transarterial infusion chemotherapy (TACE/TAI) and microwave ablation (MWA), in breast cancer and demonstrated promising local tumor control (7, 8). Locoregional interventional therapies have also shown favorable clinical efficacy, particularly in hepatocellular carcinoma (9, 10). Previous studies have shown that TAI/hepatic arterial infusion chemotherapy (HAIC) can rapidly reduce tumor burden and improve local tumor control while modulating the tumor immune microenvironment. Combined with MWA, synergistic effects may further enhance local control and reduce recurrence risk (10). Therefore, we applied this locoregional combination strategy in a patient with TNBC who was unsuitable for surgery and unable to tolerate systemic chemotherapy. Short-term postoperative adjuvant therapy was administered because of high-risk recurrence factors, resulting in durable disease control and providing a potential therapeutic option for this special patient population.

## Case presentation

**Figure 1:**
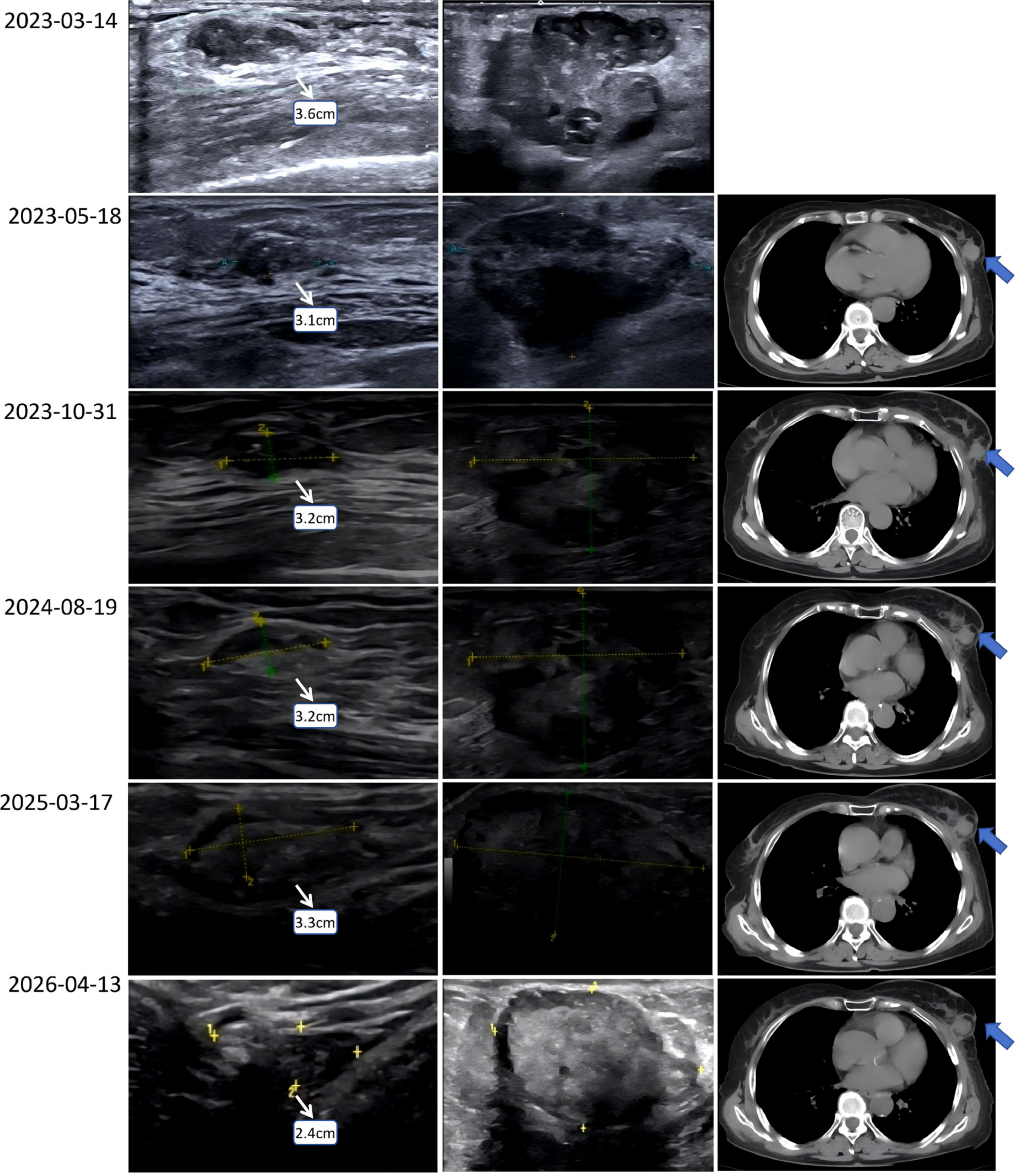
Imaging during the treatment course. Each corresponding row represents follow-up assessments at baseline, 2 months, 6 months, 1 year, 2 years, and 3 years after treatment, including breast ultrasonography and chest CT. The first column shows post-treatment changes in the right breast, left breast, and chest CT of the breast lesions.

**Figure 2:**
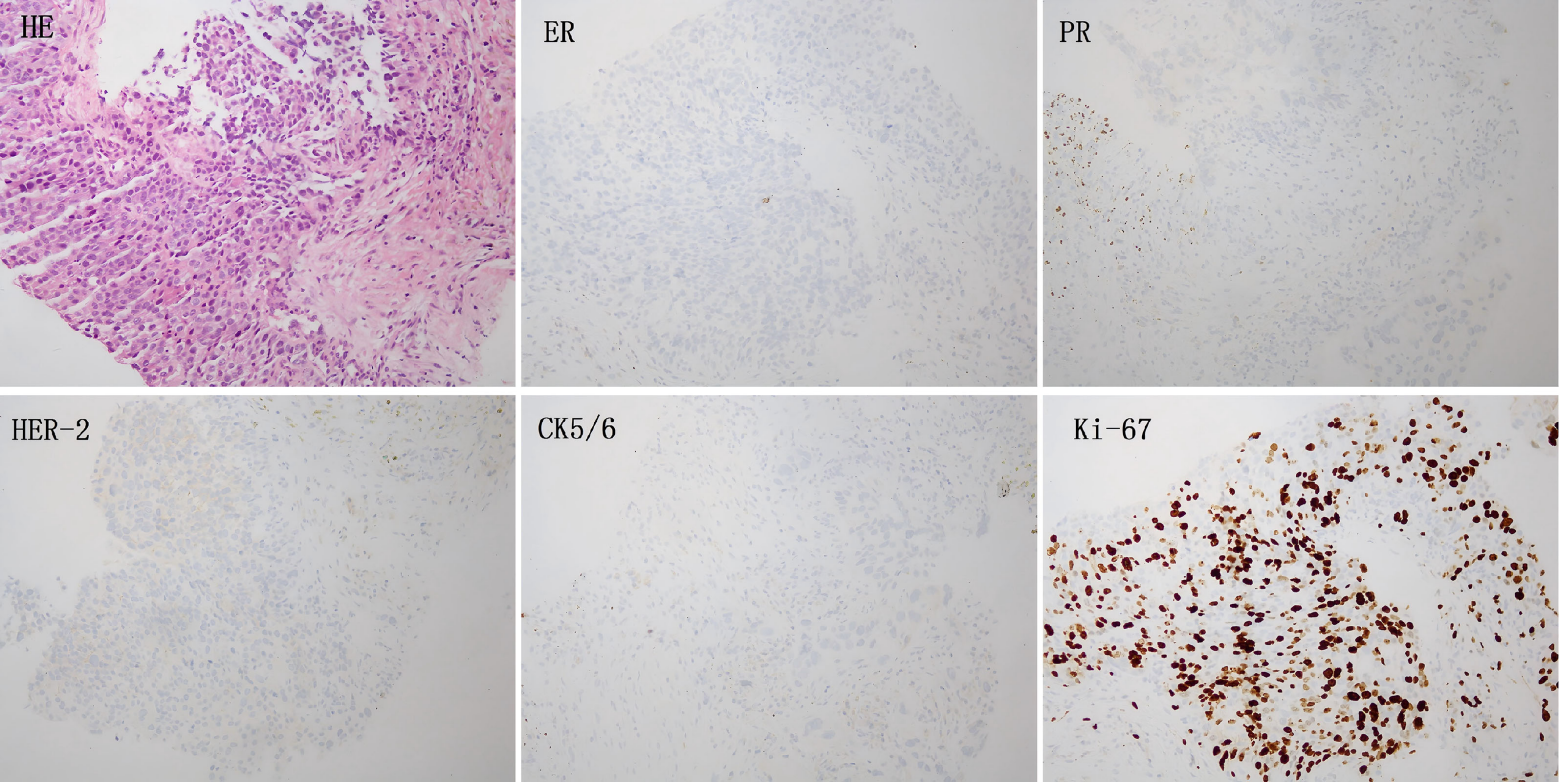
Hematoxylin and eosin (H&E) staining and immunohistochemical: ER (−), PR (−), HER2 (−), Ki-67 (approximately 50%), p53 (+), CK5/6 (−), and p63 (focal +).

A 73-year-old woman presented on March 14, 2023, with bilateral breast masses for 3 days, predominantly in the left breast. The patient had a history of cerebral infarction and coronary atherosclerotic heart disease, with cardiac function classified as New York Heart Association (NYHA) class II. The breast masses were incidentally detected during bathing. Due to multiple comorbidities and intolerance to surgery, the patient was referred to our department for further treatment. Physical examination revealed a Karnofsky Performance Status (KPS) score of 70. A 4 × 3 cm firm mass with limited mobility was palpable in the lower outer quadrant of the left breast. Serum CA15–3 and carcinoembryonic antigen (CEA) levels were within normal ranges. Breast ultrasonography demonstrated a 36 × 27 mm hypoechoic lesion in the lower outer quadrant of the left breast with indistinct margins, irregular morphology, heterogeneous internal echogenicity, and multiple anechoic areas without abundant blood flow signals. A 11.1 × 4.6 mm hypoechoic lesion was also identified in the right breast (**Figure 1**). No enlarged lymph nodes were detected in the bilateral cervical or supraclavicular regions. Chest and whole-abdominal computed tomography (CT) showed no evidence of distant metastasis. Core needle biopsy was performed for bilateral breast lesions. The right breast lesion was considered adenosis, whereas the left breast lesion was diagnosed as invasive ductal carcinoma(IDC). Immunohistochemistry showed ER (−), PR (−), HER2 (−), Ki-67 (approximately 50%), p53 (+), CK5/6 (−), and p63 (focal +) (**Figure 2**).

## Treatment and follow-up

**Figure 3:**
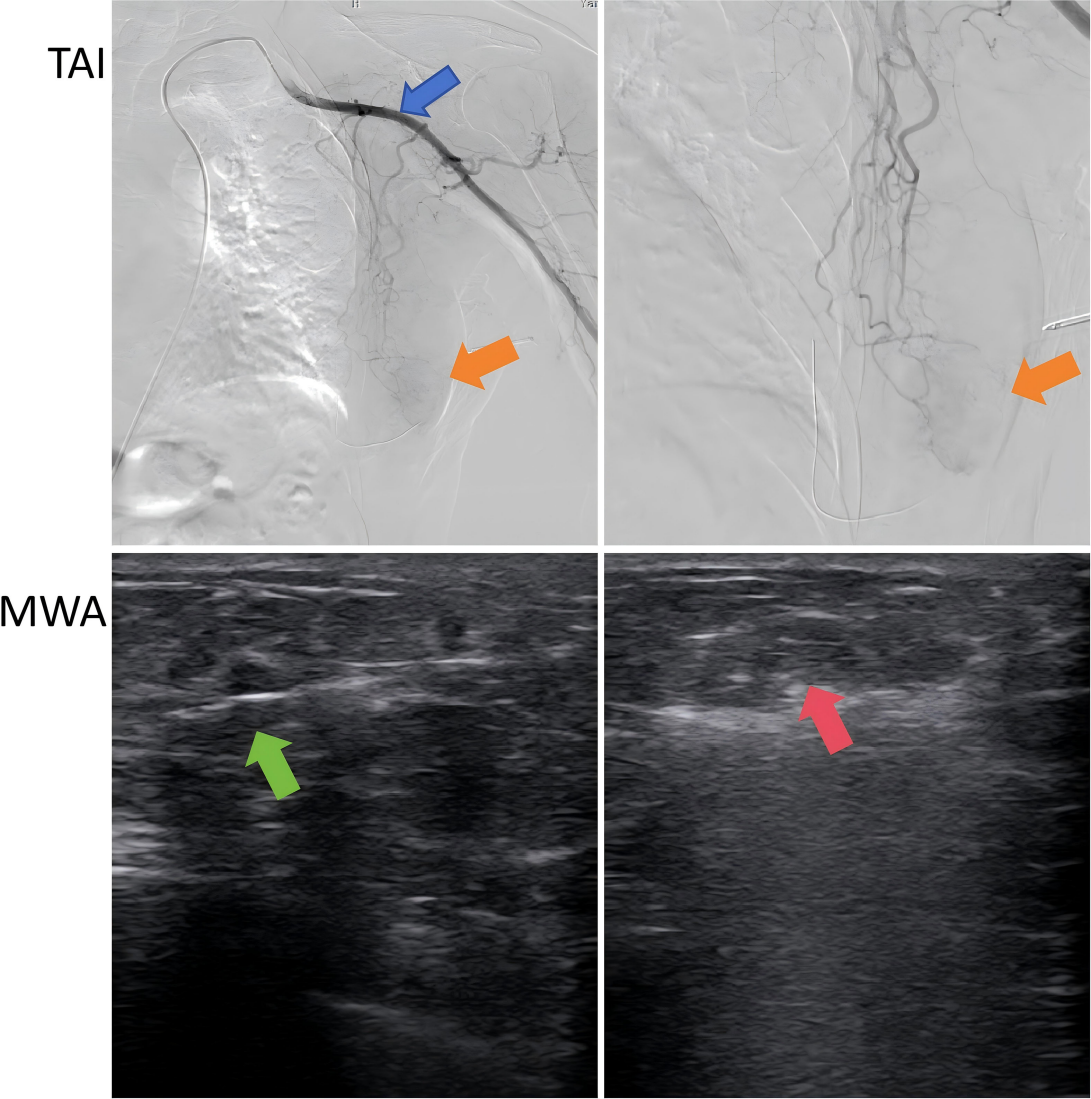
Imaging during TAI and MWA. During transarterial infusion (TAI), angiography demonstrates superselective catheterization of the lateral thoracic artery with chemotherapeutic infusion. During microwave ablation (MWA), intra-procedural and immediate post-procedural images are shown, including ultrasound-guided antenna placement and post-ablation imaging. Blue, orange, green, and red arrows indicate the lateral thoracic artery, tumor, ablation antenna, and post-ablation tumor image, respectively.

**Figure 4:**
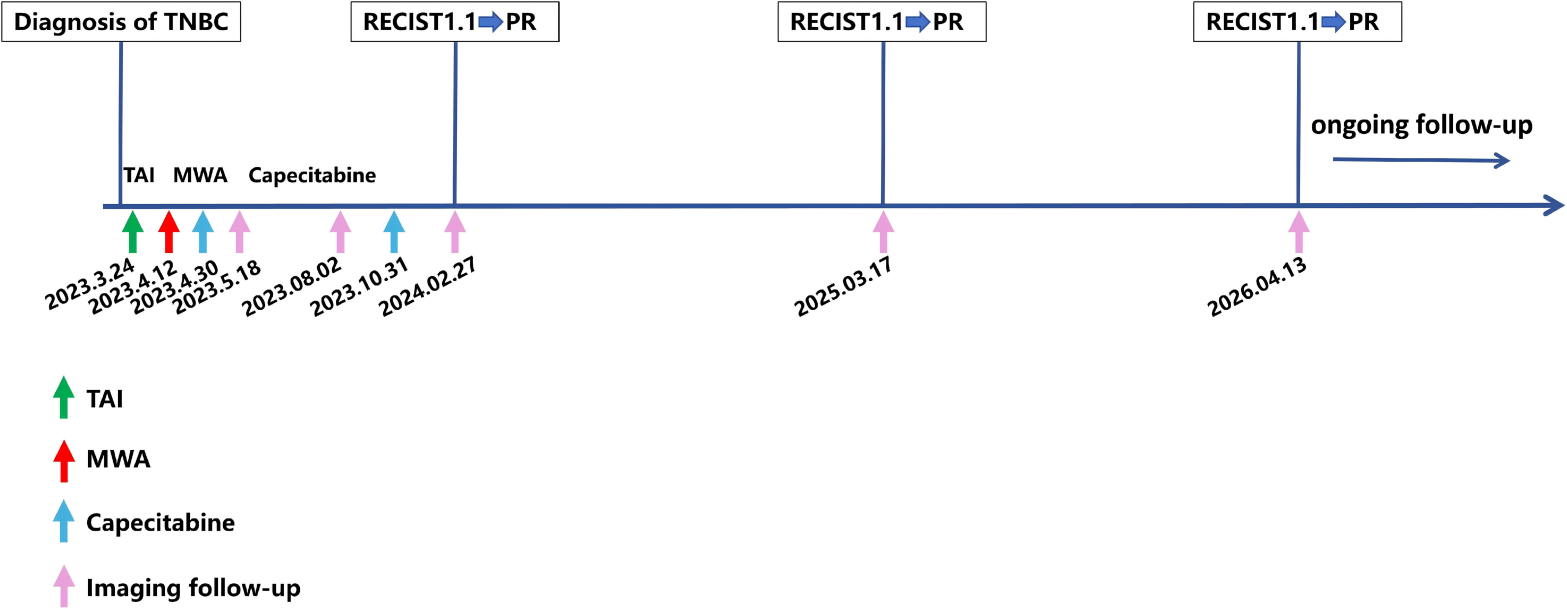
Flowchart of the treatment process.

Following multidisciplinary team (MDT) discussion, the patient was considered unable to tolerate surgery or systemic chemotherapy because of multiple comorbidities and poor overall condition. In addition, anthracycline-based intravenous chemotherapy was considered unsuitable because of its potential cardiotoxicity. Therefore, locoregional treatment was recommended. For neoadjuvant treatment purposes, transarterial infusion chemotherapy with docetaxel (60 mg) and cisplatin (60 mg) was administered through super selective catheterization of the lateral thoracic artery based on pharmacological characteristics. However, the patient developed grade 3 adverse events, including nausea, vomiting. Despite antiemetic therapy and optimal supportive care, the patient still could not tolerate the next cycle of TAI. Ultrasound-guided MWA of the breast lesion was subsequently performed 2 weeks later (**Figure 3**). Considering the high risk of recurrence in TNBC and locoregional treatment alone, the patient received adjuvant local radiotherapy and 6 months of oral capecitabine. During treatment, grade 3 adverse events (nausea, vomiting, fatigue) occurred. At 3 months, breast ultrasonography showed a reduced left breast lesion (2.9 × 1.5 cm). At the last follow-up on April 13, 2026, the patient maintained a partial response per RECIST 1.1, with a residual lesion of 2.4 cm and well-defined margins. The patient is currently alive and under follow-up without disease progression, with an ECOG performance status of 0–1 and a good quality of life (**Figure 4**).

## Discussion

Treatment response in early-stage TNBC is a critical prognostic factor (11). For patients unable to tolerate surgery and systemic chemotherapy, combined locoregional radical treatment with low-intensity systemic therapy may have potential clinical value. In this case, transarterial infusion chemotherapy followed by local ablation was used as a neoadjuvant-like locoregional strategy, with short-course adjuvant therapy to improve tumor control and reduce recurrence risk.

Locoregional interventional therapies have demonstrated promising efficacy across multiple solid tumors. In early-stage breast cancer, exploratory studies have also evaluated the application of TACE and MWA. As a potentially curative modality, MWA has shown substantial clinical benefit in various solid malignancies (11–13). Notably, evidence from hepatocellular carcinoma suggests that its efficacy may be comparable to that of radical surgical resection (13). In studies of breast cancer ablation therapy, most enrolled patients had early-stage disease with tumor diameters ≤2 cm, and MWA has been demonstrated to be a feasible treatment modality (7, 14, 15). A single-center, small-sample retrospective study reported that, over a median follow-up of 26.7 months, MWA achieved tumor control outcomes comparable to nipple-sparing mastectomy. However, MWA can be performed under local anesthesia with reduced intraoperative bleeding, indicating a more favorable safety profile and potential advantages in elderly patients with compromised performance status (7). In contrast, the application of TACE/TAI in breast cancer has predominantly been reported in case reports or studies involving breast cancer liver metastases, yet it has demonstrated favorable local control rates (8, 16–18). TAI not only delivers high intratumoral concentrations of cytotoxic agents directly to the tumor microenvironment, thereby mitigating systemic toxic exposure, but also indirectly modulates the tumor immune microenvironment, partially reversing immunosuppression (19–21). Meanwhile, MWA induces coagulative necrosis via thermal ablation, facilitating tumor antigen release and eliciting local immune activation as well as systemic antitumor immune responses (22, 23). These two modalities may exert synergistic effects, thereby potentiating the efficacy of locoregional radical therapy.

Current guidelines recommend that patients with high-risk TNBC should receive neoadjuvant therapy (24). In this case, the tumor measured 3.6 cm in maximum diameter and was located close to the skin. Although no lymph node metastasis was detected, the patient remained at high risk of distant dissemination. In addition, the close proximity of the tumor to the skin may limit the effectiveness of MWA (25). Although MWA provides locoregional control comparable to surgical resection, it does not address lymph node involvement or potential micro metastatic disease. Therefore, drawing on the neoadjuvant treatment paradigm, TAI was planned for 4–6 cycles with the aim of reducing tumor burden, controlling occult micro metastases, and decreasing recurrence risk. Cyclophosphamide is a prodrug that requires hepatic metabolism to generate its active metabolites, which represents a limitation of TAI, as not all agents are suitable for arterial infusion. Therefore, docetaxel and cisplatin were selected instead. These agents induce tumor cell apoptosis through microtubule inhibition and DNA cross-linking, respectively, and can exert rapid antitumor effects at high local concentrations. However, due to the patient’s limited tolerance to chemotherapy-related adverse events, subsequent infusion chemotherapy was discontinued. Accordingly, MWA was promptly performed as a complementary locoregional radical intervention, partially offsetting the potential impairment in local disease control associated with early termination of TAI. TNBC is characterized by high invasiveness and a propensity for early metastasis, with treatment response being highly prognostic; therefore, enhanced local control may be of critical importance in improving outcomes. As of yet, there have been no reports on the use of combined TAI and MWA as a curative approach for early-stage TNBC. This study, based on established experience in hepatocellular carcinoma and other solid tumors, represents the first application of this combined strategy in a patient with TNBC, offering a potential alternative therapeutic approach for patients who are unable to tolerate standard systemic therapy and surgical treatment. In this case, the combined strategy achieved favorable local disease control. Although TAI was discontinued due to chemotherapy intolerance during the infusion phase, immediate subsequent MWA as a complementary locoregional radical treatment resulted in durable disease control, suggesting that locoregional therapy may represent a feasible option in selected patients.

In this case, locoregional intensified therapy followed by short-course adjuvant treatment may offer a potential option for patients with limited treatment tolerance. However, TAI was not completed due to adverse events, which may have affected the overall outcome. In patients who can tolerate treatment, completion of the planned TAI course may improve clinical outcomes. No significant procedure-related adverse events were observed. Nevertheless, this study is a single case report, and the level of evidence is limited, which precludes generalization of the findings. In addition, a systematic mechanistic analysis of the tumor microenvironment was not performed. Although durable disease control was achieved, larger prospective studies are needed to validate its efficacy and safety.

## Conclusion

In conclusion, we report an elderly patient with early-stage TNBC who was unable to tolerate surgery and systemic chemotherapy. After MDT discussion, TAI followed by MWA was performed, with postoperative radiotherapy and capecitabine. The patient has remained disease-free for over 3 years. This case suggests a potential safe locoregional strategy for such patients, but further high-quality evidence is needed for validation.

## Data Availability

The original contributions presented in the study are included in the article/supplementary material. Further inquiries can be directed to the corresponding author.
